# Informing training needs for the revised certified clinical data manager (CCDM^TM^) exam: analysis of results from the previous exam

**DOI:** 10.1093/jamiaopen/ooac010

**Published:** 2022-02-22

**Authors:** Tremaine Brueon Williams, Carsten Schmidtke, Kevin Roessger, Vicki Dieffenderfer, Maryam Garza, Meredith Zozus

**Affiliations:** 1 Department of Biomedical Informatics, University of Arkansas for Medical Sciences, Little Rock, Arkansas, USA; 2 Department of Human Resource & Workforce Development, University of Arkansas, Fayetteville, Arkansas, USA; 3 Department of Adult and Lifelong Learning, University of Arkansas, Fayetteville, Arkansas, USA; 4 Department of Population Health Sciences, Joe R. and Teresa Lozano Long School of Medicine, University of Texas Health Science Center San Antonio, San Antonio, Texas, USA

**Keywords:** clinical research data management, training, informatics, competency

## Abstract

**Objective:**

To inform training needs for the revised Certified Clinical Data Manager (CCDM^TM^) Exam.

**Introduction:**

Clinical data managers hold the responsibility for processing the data on which research conclusions and regulatory decisions are based, highlighting the importance of applying effective data management practices. The use of practice standards such as the Good Clinical Data Management Practices increases confidence in data, emphasizing that the study conclusions likely hold much more weight when utilizing standard practices.

**Methods:**

A quantitative, descriptive study, and application of classic test theory was undertaken to analyze past data from the CCDM^TM^ Exam to identify potential training needs. Data across 952 sequential exam attempts were pooled for analysis.

**Results:**

Competency domain-level analysis identified training needs in 4 areas: design tasks; data processing tasks; programming tasks; and coordination and management tasks.

**Conclusions:**

Analysis of past CCDM^TM^ Exam results using classic test theory identified training needs reflective of exam takers. Training in the identified areas could benefit CCDM^TM^ Exam takers and improve their ability to apply effective data management practices. While this may not be reflective of individual or organizational needs, recommendations for assessing individual and organizational training needs are provided.

## Lay Summary

This study analyzed past data from the Certified Clinical Data Manager^TM^ Exam (CCDM^TM^) to identify potential training needs. Data across 952 sequential exam attempts were pooled for analysis. The study identified training needs in 4 areas of clinical data management: design tasks; data processing tasks; programming tasks; and coordination and management tasks. Training in the 4 identified areas could benefit CCDM^TM^ Exam takers and improve their ability to apply effective data management practices.

## BACKGROUND AND SIGNIFICANCE

Clinical data managers undertake or oversee the collection and processing of data from clinical trials:^1^ significantly distinct from the operational management of clinical data. While clinical research informatics focuses on the science supporting the management and use of data in the design, conduct, and reporting of clinical studies across the translational spectrum,^2^^,^^3^ clinical data management (CDM) offers an applied focus on the practice of managing data from clinical studies and doing so in compliance with regulations related to drug safety and efficacy approval.^1^ Scientific concepts and principles can provide professional clinical data managers with systematic methods that broadly apply across types of data and therapeutic areas and can guide the design of data management operations for a clinical study.^4^ As the CDM profession and the scientific disciplines of informatics and data science continue to mature, the CDM profession will likely increasingly leverage clinical research informatics and data science in conceptualizing the CDM profession and training programs for clinical data managers.

The quality of data from clinical studies can impact research results and regulatory decision-making. For example, pharmaceutical clinical trials are designed to test the safety and efficacy of drugs, including the frequency of adverse drug reactions that pose a potential threat to human participants.^5^ According to the Food and Drug Administration (FDA), an estimated 106 000 deaths occur annually as a result of adverse drug reactions in humans.^6^ Given that clinical data managers are responsible for managing data on a human subject’s adverse reaction to a drug in a clinical trial, there is a significant need to ensure that clinical data managers are effectively trained in handling safety data. A clinical data manager’s use of minimum standards and evidence-based best practices may reduce the number of errors in clinical trial data and yield better data for regulatory decision-making.

The use of established CDM competencies should help individuals grow their competency to the full breadth of CDM professional practice and demonstrate that achievement for potential employers. As the professional association for CDM, the Society of Clinical Data Management (SCDM) has provided professional standards for individuals who manage data from clinical trials. SCDM maintains a set of formal competencies for the CDM profession.^7^ The competencies also support the SCDM *Certified Clinical Data Management Exam*^TM^ (CCDM^TM^).^7^ The CCDM Exam^TM^ is a 1-time exam, renewable every 3 years with a minimum of 1.8 hours of appropriate continuing education units. Applicants must fulfill 1 of the following criteria: (1) hold a bachelor’s degree or higher with a minimum of 2 years full-time experience in CDM, (2) hold an associate’s degree with a minimum of 3 years of full-time experience in CDM, (3) possess 4 or more years of full-time experience in CDM, or (4) possess part-time work experience that is greater than or equal to the full-time equivalent of the above criteria. Beyond certification, clear articulation of the competencies for CDM may help organizations understand the training needs of the profession and individuals as they pursue professional development.

Historically, fundamental differences in the disciplines of CDM and clinical research informatics have fueled significant separation of the training and professional development. Discussions regarding formally bridging the 2 communities are relatively recent.^7^^,^^8^ Clinical data managers have traditionally used on-the-job and organization-based training as a primary source of acquiring professional competencies.^8^ With such a unique combination of skills required of clinical data managers and a lack of directly applicable degree programs, on-the-job and organization-based training naturally evolved as the primary means of workforce development. By using on-the-job and organization-based training as a mechanism for acquiring the competencies of the profession, clinical data managers’ knowledge of the underlying theories and concepts vary because employers generally develop training based on the needs of the clinical studies they sponsor or otherwise support without delving into underlying principles or theory. As such, the skills, knowledge, and abilities may not translate well to new organizations, types of data, or new therapeutic areas.

Toward helping clinical data managers build more generalized knowledge, skills, and abilities, over the last decade, SCDM has increased training offerings through webinars and multiweek on-line courses. The society has endeavored to align the training with certification competencies and the Good Clinical Data Management practice standards. As a result of the clear articulation of the competencies and aligned training, clinical data managers might acquire these skills more effectively, and health-related academic programs in informatics and data science may leverage the authoritative competency set in curriculum development.

Characterizing and sharing information about types of questions missed by individuals who took the CCDM *Exam*^TM^ presents an opportunity to better target professional society training towards the needs of clinical data managers. As such, this study sought to identify how individuals taking the CCDM^TM^ Exam performed in each of the CDM professional competencies tested on the exam.

## METHODS

### Study design

The University of Arkansas’ Institutional Review Board reviewed and approved this study (Protocol# 1803110198). This study employs a quantitative descriptive research design using data from past CCDM^TM^ Exam attempts. Classical test theory was applied to identify areas of competency deficit as measured by incorrect responses to questions on the exam. Classical test theory is a psychometric approach derived from the early work in classical true-score theory^9^ and was chosen for this study because of its consistent use in medical education assessments.^10^ The fundamental principle of classical test theory is that a person’s observed test score is equal to the person’s true score plus the possibility of a random error.^11^ The classical test theory analysis used here entailed a comparison between a point-biserial correlation to assess question reliability with respect to exam performance and descriptive *P*-values (percent of data managers who correctly answered an exam question) to assess question difficulty.^12^ Exam questions with descriptive *P*-values of less than 0.3 represent items that are very difficult, while questions with descriptive *P*-values above 0.8 should be interpreted as very easy.^13^ A question with a point-biserial correlation value above 0.15 indicates that exam takers answering the question correctly also performed well on the entire exam, and that exam takers answering the question wrong also scored low on the overall exam. Plotting exam items on this 2-dimensional space to distinguish between poorly performing questions and competency deficiencies is an established method of assessing exam item performance.

### Certification exam composition

The exam consisted of 130 questions. Each question on the exam was in multiple-choice format with 4 response options. Each exam question was composed of content from 1 of the 6 tested competency domains (design tasks, data processing tasks, programming tasks, testing tasks, coordination and management tasks, and review tasks) and has only 1 correct answer. Two additional competency domains identified by SCDM (training tasks and personnel management tasks) were not tested by the certification exam and thus were not addressed in this study. Each exam question is formally linked to a competency and each competency is associated with a competency domain by SCDM’s Item Review Panel, a committee of certified clinical data managers [ensuring content validity]. A clinical data manager’s response to an exam question was intended to represent their level of competence in a competency and as such, in 1 or more areas of a competency domain. To protect the integrity of the propriety certification exam, each of the exam’s 130 questions was randomly assigned a numerical identifier ranging from 1 to 130 for the analysis. Additionally, access to the exam’s deidentified data was restricted to only authors who served as members of the SCDM’s Exam Review Committee.

### Data collection and selection criteria

The deidentified data set was comprised of clinical data managers who had taken the exam in the time period from January 1, 2011 through December 2017. Thus, the subject selection was based on SCDM eligibility criteria to take the exam. These included clinical data managers who had (1) been awarded a bachelor’s degree with a minimum 2 years of full-time experience in CDM, (2) been awarded an associate’s degree with a minimum 3 years of full-time experience in CDM, and/or (3) had a minimum of a least 4 years of full-time experience in CDM (regardless of a degree). The data set included question-level data on a total of 952 exam attempts. All clinical data managers within this study were tested on the same exam questions.

### Variables and data analysis

The data were analyzed using IBM SPSS Statistics (IBM.com). For this study, questions with corrected point-biserial correlation values greater than 0.15, that is, reliable, and descriptive *P*-values of less than 0.50 were considered performing well and included in the analysis. A descriptive *P*-value of; for example, “0.3,” “0.4,” or even “0” represented the percentage of exam takers answering a question correctly. The lower the descriptive *P*-value, the more difficult the question. Exam questions with a high reliability (point-biserial correlation) and a high percentage of incorrect responses to a question (descriptive *P*-value) may indicate a need for additional training in the competency associated with the exam question. The corrected point-biserial correlation is the traditional point-biserial correlation but excludes the results of the question at the focus of the correlation.^14^ The corrected point-biserial correlation was calculated instead of the traditional point-biserial correlation because the corrected point-biserial correlation accounts for the potential overstatement of the correlation when 1 variable is partially determined by another.^15^ This was the case here given that this study’s total exam score (dependent variable) was partially determined by a clinical data manager’s response to each question (independent variable).

## FINDINGS

The test attempts provided individual responses to each of the exam’s 130 questions from 952 exam attempts. Less than 5% of clinical data managers had multiple attempts at the exam. All exam attempts were treated as unique contributions to understanding the competency domains and do not alter the conclusions. As a result, the data set provided 123 760 responses to the exam questions. The highest total exam score was 121 and the lowest total exam score was 46 with a range of 75. The mean total exam score was 91.90, the median was 93, the mode was 92, and the standard deviation from the mean exam score was 13.94. The exam data were approximately normally distributed with skewness of −0.571 (standard error of 0.079) and kurtosis of 0.071 (standard error of 0.158).

Content validity testing previously performed by SCDM in the exam development process indicated that the exam fairly assessed the competencies. To confirm that this relationship was maintained over the course of the exam administration, Cronbach’s alpha was calculated and yielded a value of 0.88 for the overall certification exam. Cronbach’s alpha provided an indication of the extent to which a clinical data manager’s total exam score (observed score) variance on the overall exam was credited to the variance of the true score.^16^ Because the Cronbach’s alpha of 0.88 was well above the threshold value of 0.70,^17^ we have confirmed that the certification exam questions do consistently measure the competence of the clinical data managers.

**Figure 1.**
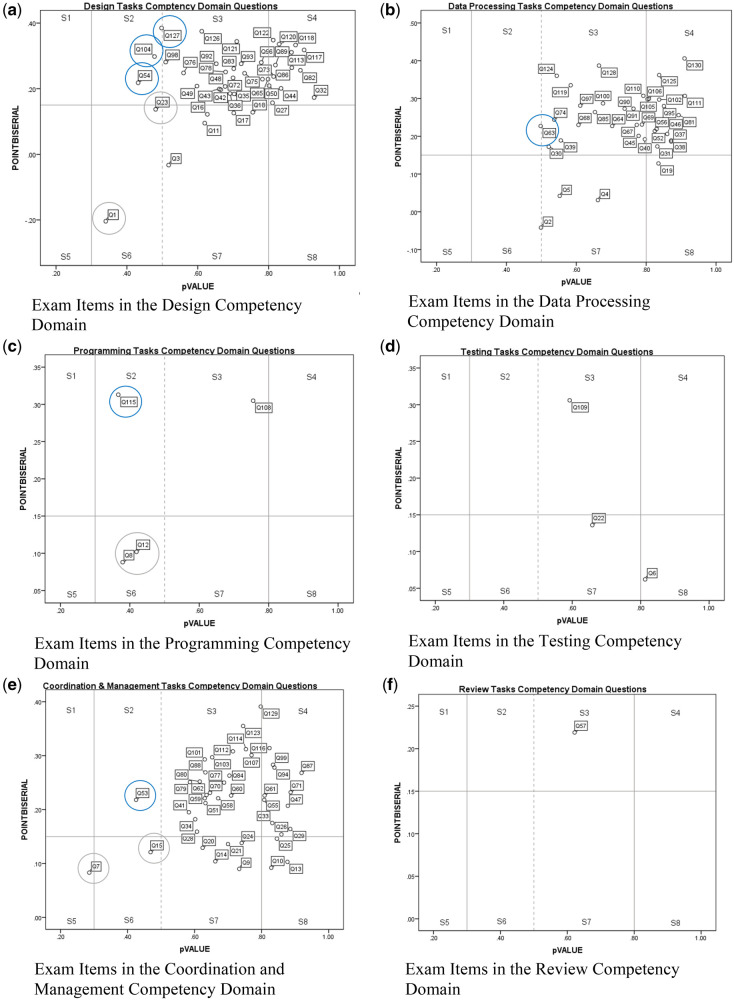
Exam item analysis plots.

Six scatterplots (Figure 1), representing the 6 tested competency domains show each question’s descriptive *P*-value and point-biserial correlation value on the x-axis and y-axis, respectively. The vertical axis of each scatterplot is divided into sections based on Kehoe’s (1995) recommendations for which the appropriate questions have descriptive *P*-values ranging between 0.3 and 0.8, with the most optimal descriptive *P*-value of 0.5, reflecting Kline’s (2005) criteria. The horizontal axis of each scatterplot distinguishes questions with point-biserial correlation values of less than 0.15, that is, those lacking reliability with respect to an individual’s performance on the rest of the exam. The 8 segments of each scatterplot (Figure 1, legend Table 1) reflect potential reasons why an exam taker may have struggled with an exam question.

**Table 1.** Legend for scatterplot figures

Segment description	Difficulty (descriptive *P*-value)	Reliability (cPBC)	Development opportunity
Segment 1 (S1)—The low descriptive *P*-value indicates a low proportion of exam takers answered correctly, that is, high difficulty. The high point-biserial correlation indicates a high level of question reliability with respect to performance on the rest of the exam. Falling in S1 indicates opportunity for additional exam preparation or other interventions in the competency associated with the question.	High (<.3)	High (>0.15)	Yes
Segment 2 (S2)—The lower descriptive *P*-values indicate more difficult questions. The high point-biserial indicates high reliability with respect to performance on the rest of the exam. We interpret questions falling in S2 as indicating opportunity for additional exam preparation or other interventions in the competency associated with the question	Moderate (.3–.5)	High (>0.15)	Yes
Segment 3 (S3)—Exam questions with point-biserial correlation values greater than 0.15 and descriptive *P*-values between than 0.5 and 0.8. The high descriptive *P*-value indicates a large percentage of exam takers correctly responded to the question. The high point-biserial correlation value indicates a high level of question reliability. Falling into S3 does not indicate appreciable gain from additional exam preparation in the competency associated with the question.	Low (.5–.8)	High (>0.15)	No
Segment 4 (S4)—The high descriptive *P*-value indicates a large percentage of exam takers correctly responded to the question. The high point-biserial correlation value indicates a high level of question reliability. Falling into S4 does not indicate appreciable gain from additional exam preparation in the competency associated with the question.	Very low (.8–1.0)	High (>0.15)	No
Segment 5 (S5)—S5 contains the most difficult (lowest descriptive *P*-values) and least reliable (lowest point-biserial correlation) questions. These statistics show that many exam takers struggled with these questions. A conclusion regarding whether additional training or other exam preparation in this area may be of value to future test takers could not be reached because of the coexisting low reliability.	High (<.3)	Low (<0.15)	Inconclusive
Segment 6 (S6)—A question falling in S6 indicates that some test takers struggled with the question because of the lower descriptive *P*-value. A conclusion regarding whether additional training or other exam preparation in this area may be of value to future test takers could not be reached because of the coexisting low reliability.	Moderate (.3–.5)	Low (<0.15)	Inconclusive
Segment 7 (S7)—A question falling in S7 indicates that test takers did not appreciably struggle with the question. Similarly, no need for additional exam preparation in the competency associated with the question is indicated. However, the low reliability indicates that the question itself could be improved for future exam versions.	Low (.5–.8)	Low (<0.15)	No
Segment 8 (S8)—Questions falling in S8 have the largest percentage of correct responses indicating that most test takers did not appreciably struggle with the questions. Similarly, no need for additional exam preparation in the competency associated with the question is indicated. However, the low reliability indicates that the question itself could be improved for future exam versions.	Very low (.8–1.0)	Low (<0.15)	No

cPBC: corrected point-biserial correlation.

The percentage of correct responses differed by the domain (Table 2). The 3 competency domains with high relative numbers of questions all had higher than a 70% correct response rate to exam questions in the competency domain. The 3 domains with less than 5 questions received significantly lower proportions of correct responses and had a much higher percentage of questions below the reliability limit.

**Table 2.** Percentage of correct responses by competency domain

Competency domain	Number of questions in competency domain^a^ (over reliability limit)	Average percent correct overall (95% CI)	Average percent correct for questions over reliability limit (95% CI)
Design tasks	42 (35)	70.1% (69.6–70.5)	72.5% (72.1–73.0)
Data processing tasks	37 (33)	73.2% (72.7–73.6)	74.3% (73.8–74.8)
Programming tasks	4 (2)	48.0% (46.4–49.6)	56.1% (53.8–58.3)
Testing tasks	3 (1)	68.8% (67.1–70.5)	59.2% (56.0–62.4)
Coordination and mgt. tasks	43 (33)	71.1% (70.6–71.5)	72.1% (71.6–72.6)
Review tasks	1 (1)	62.2% (59.0–65.3)	62.2% (59.0–65.3)

Number of questions per competency domain on the exam version analyzed.

CI: 95% confidence intervals.^18^

With respect to the 130 individual questions and the competencies with which they were associated, 6 competencies above the 0.15 reliability had less than 50% of the exam takers answering incorrectly (Table 3). These were the 6 competencies where additional training would add value to exam takers.

**Table 3:** Identified training needs

Competency domain	Individual competency		Descriptive *P*-value	Corrected point-biserial
Design tasks	Implementing data standards	0.06^a^	.4317	0.218
Design tasks	Specifying edit checks	0.08^a^	.4779	0.298
Design tasks	Designing data collection forms	0.04^a^	.4979	0.385
Data processing tasks	Entering data	0.02^a^	.4979	0.227
Programming tasks	Programming data extracts	<0.01^a^	.3666	0.313
Coordination and management tasks	Coordinating data discrepancy identification and resolution	0.06^a^	.4254	0.218

Percentage of total examine questions (*n* = 130) per each individual competency.

## DISCUSSION

### Summary of overall findings

Analysis of 952 attempts at the CCDM^TM^ Exam indicated several areas where additional training may benefit clinical data managers (Table 3). At the domain level, training needs were identified in 4 competency domains (ie, design tasks; data processing tasks; programming tasks; coordination and management tasks): providing additional implications within the question-level analysis. The development and use of additional training in the 4 competency of the exam may improve exam performance and job performance. However, job performance is not based solely on command of the required knowledge or an exam score. Job performance acknowledges the potential impact of other variables that could increase or decrease success, many of which are external.^19^ These factors could include tools needed to execute a job, the design of a job task, or the presence and use of job aids such as checklists, decision support, automation, written procedures, or structured forms.^20^ Within the context of training needs and job performance, the domain-level, and question-level findings are discussed.

### Domain-level findings

The domain-level analysis identified training needs in 4 competency domains: design tasks; data processing tasks; programming tasks; and coordination and management tasks. Based on the reliability results (Table 2), these 4 domains contained the minimum number of questions that were required to support training need identification. For example, the exam contained 42 certification exam questions that were within the design tasks competency domain. This contained 35 questions above what was required for sufficient domain-level reliability.

The design competency domain had an average percentage of correct responses slightly lower than the data processing and coordination and management domains. While the 95% confidence intervals do not overlap, the difference is likely not meaningful in practice.

The 3 remaining competency domains (ie, programming, testing, and review) had a significantly lower proportion of questions over the reliability limit and a significantly lower proportion of correct responses. Given the low number of questions in each of these 3 competency domains, the domains were not covered enough by the exam questions to effectively support an investigation of whether training needs existed within these domains. For example, the single competency in the review domain encompassed the tasks of the reviewing of clinical study protocols, that is, to identify and interpret information needed to set-up information systems to support studies and manage study data. However today, the review domain includes competencies associated with the review of study data represented in tables, listings, figures, and clinical study reports for the purpose of verifying that these representations accurately reflect the data collected, and for review of data management work performed by others. Given the large number of exam attempts in the data set, the lower scores, while likely not reflective of the domain may indeed reflect lower performance on the individual competencies. This limits domain-level interpretation of the results from these 3 domains. The 3 domains with the majority of exam questions likely had more complete domain coverage, and thus the domain analysis is more likely reflective of performance within the domain area.

### Question-level implications

When further interpreted at the question level, these 4 training needs indicated potential improvements in 6 individual CDM competencies (ie, implementing data standards; specifying edit checks; designing data collection forms; entering data; programming data extracts; coordinating data discrepancy identification and resolution). While each of these 6 competencies met the statistical criteria of a training need, a clear distinction could not be made as to whether the issue was a problem with the specific exam question or whether it reflected more generalized lack of knowledge of the competency.

Five of the 6 individual competencies (ie, implementing data standards; specifying edit checks; designing data collection forms; entering data; coordinating data discrepancy identification and resolution) accounted for 2%–8% of the total exam questions. Broadly, each of the individual competencies should have had a sufficient number of questions so that the training needs within individual competencies could have been more effectively assessed.^10^^,^^13^^,^^14^ Only 1 exam question per individual competency met the descriptive *P*-value and cPBC statistical criteria. If additional exam questions that tested these individual competencies had met the statistical criteria, a clearer indication of a training need would have been provided.

Programming data extracts met the statistical criteria of a training need with a descriptive *P*-value of .3666 and cPBC of .313. However, programming data extracts accounted for <1% of the total exam questions. For programming data extracts to have been interpreted as a training need, the percentage of its exam questions should have been larger, and additional exam questions that tested this individual competency should have met the statistical criteria.

### Application of findings to future exams

The exam was previously organized into 3 sections: section 1—covering project management, process design, data management planning, form design, relational databases, and CRO-Sponsor partnership, section 2—covering processing on local and central lab data, and section 3—covering safety data management. The previous exam’s 130 questions corresponded to 130 competencies. In response to maturing of the CDM profession and in part to support future revision of the exam, SCDM undertook job analyses in 2015^8^ and 2019.^7^ As part of these job analyses, the competencies were further developed into the 8-domain framework used here and the previous 130 competencies and associated exam questions were mapped to the 67 new competencies across the new 8 competency domains. This mapping was used here so that the results of the analysis would align with the future exam. According to the most recent job analysis,^7^ the programming, training, and review competency domains have been expanded to better cover the domains to 8, 2, and 3 competencies, respectively. Though the number of competencies in these domains remains small, due to the consolidation of competencies, each competency can be allotted multiple questions on the revised exam. Thus, the number of questions per competency will also increase. Uncertainties in mapping, such as whether an old and more detailed competency belongs in a new competency and associated competency domain versus the other, limit the extent to which this analysis can be predictive of future exam performance and caution is enjoined in making such prediction. Though much of the exam is being replaced, further limiting our ability to generalize to the future revised exam, this alignment enables performance on the past exam to be more easily factored into decision-making by those preparing to take the revised exam as well as those supporting exam preparation efforts.

While aggregate results from past exams can be informative, an individual’s exam preparation should be based on areas where the individual lacks awareness or experience rather than on the aggregate performance of past exam-takers. Individuals preparing for the exam can target their preparation through self-assessment by rating their actual experience in each competency. For example, considering the competency *Specify edit checks*, and rating actual experience in categories ranging from, “I do not know what specifying edit checks is” to “I have specified edit checks for multiple studies and train others in this task.” Averaging by competency domain will identify the domains where additional exam preparation will be the most helpful. Self-assessments can be pooled for individuals within a study group to prioritize training or study topics. The method can be applied to identify training areas of priority to an individual or organization by simultaneously rating the competency according to the scale above in addition to rating each competency according to relevance to their job, or the job responsibilities at a particular organization.

With the most recent job analysis and competency set, the content alignment work within SCDM initiated in 2015 has resulted in a set of professional competencies that is surveyed and maintained over time and processes for maintenance of the CCDM^TM^ Exam to assure that the exam remains reflective of those current competencies. To the extent that this alignment is maintained and the exam competencies remain representative of the needs of organizations, the CCDM^TM^ Exam will distinguish individuals with the desired competencies from those lacking them. New degree programs seeking to develop the CDM workforce will benefit from the maintained competencies. Education and training toward CDM competencies at all levels from awareness for clinical investigators and research team members, and competencies for professional clinical data managers will improve outcomes for professionals, organizations, and those that depend on the data and results from clinical studies.

### Limitations

The proportion of exam takers outside the United States significantly increased during the time period from which data were analyzed. Thus, these pooled results may not be reflective of the needs of individuals seeking certification today or of the organizations where they work. Additionally, programming data extracts, entering data, and designing data collection forms could not be considered as training needs because they did not contain enough questions on the exam.

## CONCLUSIONS

Application of classic test theory identified training needs reflective of past CCDM^TM^ Exam takers. The competencies reflecting the highest area of training need include: implementing data standards, specifying edit checks, designing data collection forms, entering data, programming data extracts, and coordinating data discrepancy identification and resolution. The competency domains with the largest training needs included programming, testing, and review. While this observational retrospective analysis cannot be generalized to predict actual training needs for the revised exam, these results taken in the context of their limitations and in addition to self- and pooled group-assessment provide a foundation for preparation for the revised exam. Finally, these findings may also be applicable to other types of research and activities related to the secondary use of electronic health record and real-world data, including the generation of Real World Evidence for FDA-regulated products, and registries.

## FUNDING

This work was supported by the National Center for Advancing Translational Sciences of the National Institutes of Health grant numbers TR003107 and KL2 TR003108. The content is solely the responsibility of the authors and does not necessarily represent the official views of the National Institutes of Health nor Society for Clinical Data Management.

## AUTHOR CONTRIBUTIONS

TBW: Led the study group and design, coordinated data collection and analysis, and drafted manuscript. CS: Participated in study design and research protocol. KR: Participated in study design and data analysis. VD: Participated in study design and interpretation of results. MG: Participated in study design and interpretation of results. MZ: Participated in study design, interpretation of results, and manuscript revisions.

## CONFLICT OF INTEREST STATEMENT

None declared.

## DATA AVALIABILITY

The data underlying this article were provided by The Society for Clinical Data Management by permission. Data will be shared on request to the corresponding author with permission of The Society for Clinical Data Management.

## References

[1] Krishnankutty B, Naveen Kumar B, Moodahadu L, Bellary S. Data management in clinical research: an overview. Indian J Pharmacol 2012; 44 (2): 168. 22529469 10.4103/0253-7613.93842 PMC3326906

[2] Kahn M, Weng C. Clinical research informatics: a conceptual perspective. J Am Med Inform Assoc 2012; 19 (e1): 36–42. doi:10.1136/amiajnl-2012-000968. PMC3392857 22523344

[3] Embi P, Payne P. Clinical research informatics: challenges, opportunities and definition for an emerging domain. J Am Med Inform Assoc 2009; 16 (3): 316–27. 19261934 10.1197/jamia.M3005 PMC2732242

[4] Zozus MN. Letter from the editor. Data Basics 2020; 26 (2): 6–17.

[5] Edwards I, Aronson J. Adverse drug reactions: definitions, diagnosis, and management. The Lancet 2000; 356 (9237): 1255–9. 10.1016/S0140-6736(00)02799-9 11072960

[6] Preventable adverse drug reactions: a focus on drug interactions. U.S. Food and Drug Administration. https://www.fda.gov/drugs/drug-interactions-labeling/preventable-adverse-drug-reactions-focus-drug-interactions. Accessed September 5, 2020.

[7] Abouelenein S, Williams T, Baldner J, Zozus MN. Analysis of professional competencies for the clinical research data management profession. Stud Health Technol Inform 2020; 270: 1199–200. 32570578 10.3233/SHTI200361

[8] Zozus M, Lazarov A, Smith L, et al Analysis of professional competencies for the clinical research data management profession: implications for training and professional certification. J Am Med Inform Assoc 2017; 24 (4): 737–45. 28339721 10.1093/jamia/ocw179 PMC6080682

[9] Allen M, Yen W. Introduction to Measurement Theory. Monterey; CA: Brooks-Cole; 1979.

[10] De Champlain A. A primer on classical test theory and item response theory for assessments in medical education. Med Educ 2010; 44 (1): 109–17. 20078762 10.1111/j.1365-2923.2009.03425.x

[11] DeVellis R. Classical test theory. Med Care 2006; 44: 50–9. doi:10.1097/01.mlr.0000245426.10853.30. 17060836

[12] Kunovskaya I, Cude B, Alexeev N. Evaluation of a financial literacy test using classical test theory and item response theory. J Fam Econ Iss 2014; 35 (4): 516–31.

[13] Kehoe J. Basic item analysis for multiple-choice tests. Practical Assessment, Research, and Evaluation 1994; 4 (10): 1–3. doi: 10.7275/07zg-h235.

[14] Crocker L, Algina J. Introduction to Classical and Modern Test Theory. New York, NY: Holt, Rinehart and Winston; 1986.

[15] LeBlanc V, Cox M. Interpretation of the point-biserial correlation coefficient in the context of a school examination. TQMP 2017; 13 (1): 46–56.

[16] Cronbach L. Coefficient alpha and the internal structure of tests. Psychometrika 1951; 16 (3): 297–334.

[17] Salkind N. Statistics for People Who (Think They) Hate Statistics. 4th ed. Los Angeles, CA: Sage; 2016.

[18] Fleiss J, Levin BL, Paik M. Statistical Methods for Rates and Proportions. 3rd ed. New York, NY: Wiley; 2003.

[19] Wittkuhn K. Understanding performance improvement. Perf Improv 2016; 55 (6): 13–8.

[20] Rothwell W. Beyond Training and Development: The Groundbreaking Classic on Human Performance Enhancement. 2nd ed. New York, NY: American Management Association; 2005.

